# Networking among young global health researchers through an intensive training approach: a mixed methods exploratory study

**DOI:** 10.1186/1478-4505-12-5

**Published:** 2014-01-25

**Authors:** Lindsey M Lenters, Donald C Cole, Paula Godoy-Ruiz

**Affiliations:** 1 Global Child Health, The Hospital for Sick Children, 686 Bay Street, Toronto, Ontario M5G 0A4, Canada; 2 Dalla Lana School of Public Health, University of Toronto, 155 College Street, Toronto, Ontario M5T 3M7, Canada; 3 Global Health Program, Department of Family and Community Medicine, University of Toronto, 500 University Avenue, Floor 5, Room 550, Toronto, Ontario M5G 1V7, Canada

**Keywords:** Collaboration, Continuing education, Developing countries, Knowledge translation, Mixed methods, Networks

## Abstract

**Background:**

Networks are increasingly regarded as essential in health research aimed at influencing practice and policies. Less research has focused on the role networking can play in researchers’ careers and its broader impacts on capacity strengthening in health research. We used the Canadian Coalition for Global Health Research (CCGHR) annual Summer Institute for New Global Health Researchers (SIs) as an opportunity to explore networking among new global health researchers.

**Methods:**

A mixed-methods exploratory study was conducted among SI alumni and facilitators who had participated in at least one SI between 2004 and 2010. Alumni and facilitators completed an online short questionnaire, and a subset participated in an in-depth interview. Thematic analysis of the qualitative data was triangulated with quantitative results and CCGHR reports on SIs. Synthesis occurred through the development of a process model relevant to networking through the SIs.

**Results:**

Through networking at the SIs, participants experienced decreased isolation and strengthened working relationships. Participants accessed new knowledge, opportunities, and resources through networking during the SI. Post-SI, participants reported ongoing contact and collaboration, although most participants desired more opportunities for interaction. They made suggestions for structural supports to networking among new global health researchers.

**Conclusions:**

Networking at the SI contributed positively to opportunities for individuals, and contributed to the formation of a network of global health researchers. Intentional inclusion of networking in health research capacity strengthening initiatives, with supportive resources and infrastructure could create dynamic, sustainable networks accessible to global health researchers around the world.

## Background

### Networks and global health research

In health research, networks are seen to play a key role in enhancing the generation, dissemination, and use of scientific knowledge [1]. Recent decades have seen a rapid proliferation and evolution of intentional, designed networks, driven by developments in information and communication technologies as well as by globalization [2]. International [3] and global [4] health are no exceptions, with networks increasingly playing a role in coordinating health research for development and policy influence [5,6]. Closely allied are health research capacity strengthening initiatives, which are embedded in research networks [5,7], operate as networks [8], or are promoted by non-governmental organizations (NGO) that function as networks [4,9].

### Networks and networking

A network can be loosely defined as a structure linking together individual and organizational actors with shared goals or values, though often not a shared geography [10]. There is a growing interest in the role that networks play in enhancing efficiency, equity, and productivity in a wide range of contexts both in the private sector and the public sector [11]. Approaches developed to help understand the structure and functions of networks include the network functions approach [2], social network analysis [12], and the lifecycle approach [1]. These approaches tend to focus on the structure of the network, the linkages within the network, and their importance for the individuals who comprise the network [1] but focus less on the processes involved in the development and maintenance of networks [13]. Personal connections and information sharing often lead to the development of more formal networks and their success depends on the maintenance of trust, communication, and mutual support [11]. Developmental networks may extend internationally, as a form of distributed mentoring, often tailored by individuals to meet their needs [14].

Networking has been defined as “an individual’s behaviors aimed at building, maintaining and using informal relationships that possess the potential to assist them with their work or career” [15,16]. The facets, functions, and impacts of networking have been researched primarily in the context of career development and upward mobility within the private sector [13-16]. These studies have focused on correlating networking behaviors and personal attributes with both objective outcomes, such as promotions and increased compensation, as well as subjective outcomes such as career satisfaction.

A nascent literature is emerging on the process of networking in health research. As Chanda-Kapata et al. [17] note: “Networking provides proven opportunities for researchers to share their knowledge on the conduct, regulation, coordination and financing of health research – and, of course, on their findings, methodologies and syntheses. Networks can be instrumental in the diffusion of innovations, from better clinical practice to the development of new methodologies (e.g., policy briefs and dialogues) to the promotion and achievement of internationally-mandated standards.” Networking has been identified as an activity in which health professionals engage as part of their formal studies [18] and an outcome intentionally promoted as part of short-term training workshops embedded in regional health research training schemes [19].

However, questions remain about networking in global health research. How can relationships be promoted and supported? How does networking contribute to the overall enhancement of capacity for global health research? An opportunity to explore such questions among new global health researchers arose through an evaluation of an NGO-sponsored research capacity strengthening initiative, the Canadian Coalition of Global Health Research (CCGHR) Summer Institute series [20].

### CCGHR Summer Institute program

The CCGHR is a Canada-based global network promoting better and more equitable health worldwide through the production and use of knowledge [9]. In 2004, the CCGHR began an annual program to bring together partners from low- and middle-income countries (LMICs) as well as Canada, called the “Summer Institute for New Global Health Researchers” (SI). The stated intention was to strengthen partnerships, facilitate translation of research into action, and develop global health research competencies. New researchers, defined internally by CCGHR as individuals who have less than five years of research experience, from Canada and LMICs formed their own “dyads” or “triads”, coming together to work on a specific research topic, and each dyad or triad was mentored by a more experienced researcher, or “facilitator”. Each SI had a preparatory phase, a face-to-face (F2F) session, and a follow-up phase. Seven SIs were offered from 2004 to 2010, organized with a range of Canadian and Southern partners. The SIs were held in Canada and LMICs including Mexico, India, Burkina Faso, Tanzania, and Ecuador.

The SI utilized several innovative approaches, including the requirement for applications from Canadian-LMIC pairs (or dyads) of newer researchers to global health that were collaborating, or plan to collaborate on a research project. Additionally, in more recent years the dyads were expanded to triads, to promote working more directly with policy makers both in the generation and use of research. The intention behind these inter-connections was to facilitate enhanced collaboration, knowledge-translation, and the creation network of global health researchers. Over 190 participants took part in the SI program over the seven years.

In response to participants’ interest in gaining mentorship competencies, CCGHR implemented a facilitators-in-training (FIT) initiative in 2007 where, each year, two alumni became facilitators for new participants in a subsequent SI, with mentoring by a more senior facilitator. Furthermore, a SI alumni leadership program (SI-LEAD) was piloted in 2008 with the objective of providing a small number of alumni with additional opportunities to strengthen their leadership skills. Mentees were paired with a mentor to develop learning objectives to enhance their leadership skills. Eight FITs were supported and approximately fifteen alumni gained leadership training.

### Objective and research questions

Our objective in this paper was to explore the role that networking played in the various stages of the SI program and the mechanisms for network development.

We posed the following questions:

1. How do participants perceive their experience with networking at the SI?

••What forms and functions of networking do they describe?

••What were positive experiences as well as challenges to networking?

••What value is ascribed to networking within the context of research and career development?

2. Based on insights from SI participants’ perspectives and experience, how does networking contribute to the overall enhancement of capacity for global health research?

Participating alumni and facilitator perspectives, taken together with insights from the growing literature on research networking and networks, were used to develop a process model of the forms and functions of networking.

## Methods

### Overview of study design

A mixed-methods exploratory study was conducted among SI alumni, FITs, and facilitators who had participated in at least one SI between 2004 and 2010. A general framework was developed to guide the approach to the research (Additional file 1) using insight from the literature as well as the researchers’ experience and knowledge. The research was conducted in two phases, the first consisted of an online short questionnaire (n = 62) and the second of semi-structured interviews (n = 39). Phase I data was descriptively analyzed and also used to inform phase II data collection, both as a sampling frame and as a source of information to inform interview guide development. The qualitative interviews were analyzed in an iterative process of identifying emergent themes, coding, comparing, and further refinement of the coding framework by the research team [21,22]. Thematic content analysis was performed using NVivo 9® qualitative data analysis software [21-23]. Approval for this study was provided by the University of Toronto Research Ethics Board.

### Participant recruitment

Recruitment was conducted primarily via email with efforts made to include participants from all years of the SI program (2004–2010) and with a balanced mix of gender, region of origin, year of participation, level of training, and role in the SI (participant-alumnus, FIT, or facilitator). Participants were given the option to provide consent and complete the survey using the SurveyMonkey® online survey system, by email, or by verbal response over the phone with a research assistant. The email invitation, survey, and interview were available in English, French, and Spanish.

### Data collection

In phase I, 62 former SI participants completed a short questionnaire. All participants chose to complete the survey online through SurveyMonkey®. The survey included six multiple-choice questions and took 5–10 minutes to complete. The survey asked about the SI in which they participated, implementation of their work from the SI, confidence in a series of global health competencies, involvement in global health research, clinical and training activities, and whether they maintained professional and social contact with other SI participants. Surveys were tailored to language of preference and participant role (alumnus or facilitator/organizer) using skip-logic. Results were exported from SurveyMonkey® into an Excel spreadsheet, and were descriptively analyzed for patterns. At the end of the survey, participants were asked if they would consent to participation in phase II, in the form of an interview to further expand on their experiences.

Respondents to the phase I survey who consented to the phase II interview, participated in a 35–50 minute Skype or telephone interview (n = 39). Interviews were conducted by three members of the research team and were recorded with the permission of the participants. The semi-structured interview guide was developed by the research team, and informed by the literature on health research capacity development. The interview guide was piloted among CCGHR member SI participants, and modified based on research team discussions. The initial drafts were developed in English and the final interview guide was translated into French and Spanish (see final outline in Additional file 2). The interview guide had structured categories and flexible, open-ended questions within each major category.

### Analysis

Interviews were transcribed and identifying information was removed. Interviews in French and Spanish were translated into English prior to being analyzed. A flexible coding framework was developed, based on our evaluation model conceptualizing the major anticipated outcomes, and pilot-tested using five interviews to ensure reliability across the four research team members who analyzed the interviews. Each analyst kept a coding journal to track new codes, noteworthy findings and issues to be discussed. The coding framework was updated to reflect these ideas and interview content at bi-weekly team meetings. To enhance validity, all interviews were coded by two separate members of the research team. Of the four researchers who were involved in qualitative data analysis, each one analyzed and coded a set of transcripts as the ‘first’ coder and then analyzed a second set of transcripts as a ‘second’ coder. The second coder’s role was to double-check the first round of analysis and modify the coding as appropriate. Where necessary, the research team discussed and reconciled coding discrepancies via group consensus. Using NVivo 9® software, the research team conducted thematic content analysis, identifying key themes emerging from the interview transcripts.

Since the theme of networking emerged as a significant process and outcome of participation in the SI, a deeper analysis of the phase II interview transcripts was conducted. To tease out a more detailed understanding of networking in global health research, we developed a specific analysis framework built on initial findings and the networking literature (Additional file 1).

### Research team

The research team was composed of five core members. Four team members were involved in all stages of the research: a senior faculty member with extensive experience in global health research, research capacity development and, more recently, its evaluation; a junior researcher with experience in health systems and policy research in LMICs, research capacity development and its evaluation; a junior researcher with experience in qualitative global health research; and a public health master’s student with an interest in global health. Of these team members, all but the graduate student had prior exposure of the CCGHR SIs (two as FITs, one as a facilitator and co-chair). The final team member, a recent international development BA graduate, participated in the analysis phase only and was responsible for database management and initial analysis of the quantitative data. This team member had not participated in the SI program. All research team members had experience in global health and/or in international development. Given the range of research and global health experience, as well as the varied involvement in the SI program, the team was able to approach the research from a fluid range of insider and outsider positions.

## Results

### Participant response and description (from phase I data)

Among the 190 past SI participants, 62 (33%) completed the online survey. Of the latter, 81% (n = 50) agreed to participate in phase II and 12 declined: 4 alumni, 1 FIT, and 7 facilitators. Among the 50 phase II participants, 39 interviews (63%) were successfully conducted and 11 were frustrated by scheduling conflicts or non-responses to requests to schedule an interview. Special efforts were made to include alumni and facilitators from all previous SIs, so that for every SI year a minimum of five individuals participated in this follow-up study.

Many SI participants attended multiple SIs and had multiple roles such as organizer and facilitator. Respondents were therefore classified into a predominant role and the appropriate interview guide was used. The following describes respondents by their role at the SI: alumnus, participated as a trainee once; FIT was an SI alumni who became a FIT at a subsequent SI; facilitator-organizer had a recent role as a facilitator or organizer, but may have at some point also been an alumnus. See Table 1 for participation by predominant role.

**Table 1:** Participants by role at the Summer Institute and self-selected language of response, by study phase

	**Predominant Role at Summer Institute(s)**			**Total**
**Phase**	**Alumni**	**FIT**	**Facilitator/organizer**	
I	n = 36	n = 8	n = 18	n = 62
	31 English	3 English	13 English	47 English (66%)
	3 French	3 French	1 French	7 French (11%)
	2 Spanish	2 Spanish	4 Spanish	8 Spanish (13%)
II	n = 23	n = 8	n = 8	n = 39
	18 English	6 English	6 English	30 English (77%)
	3 French	1 French	1 French	5 French (13%)
	2 Spanish	1 Spanish	1 Spanish	4 Spanish (10%)

*FIT*, Facilitator-in-training.

Response rates were comparable across the 3 languages in phase II (70–80% agreement in each language category). Table 1 shows the response language for each phase. English was the most commonly selected language, though this was not necessarily reflective of “first language” nor region of origin of respondents. In phase II, 28 respondents were Canadian-based researchers and 11 were from LMICs (4 from Latin America, 6 Africa, and 1 from Asia and Oceania).

Participants all came to the SI with some connections in the field of global health research, and with a wealth of knowledge and resources based on their previous experiences and areas of expertise. Participants had to have at least some established connections, because being part of a dyad/triad was a prerequisite for acceptance into the SI program. Additionally, it was evident that prior to participating in the SI, participants were part of various networks: the majority of respondents heard about the SI through a colleague in their home institution, and several respondents’ were told about the SI by their thesis supervisor. Several respondents were familiar with coalition activities through membership in CCGHR or attendance at CCGHR events, and the remaining respondents heard about the SI through a conference, listserv, a scholarship program, or their dyad partner.

### Networking: processes, functions, and outcomes (results primarily from phase II)

#### Networking as an expectation and outcome of the SI

The theme of networking was both a prominently expressed expectation and outcome of participating in the SI: 22/39 respondents (56%) mentioned networking as something they expected to gain from participating in the SI and 26/39 respondents (67%) mentioned networking as an outcome. For many, networking was cited as the most important outcome of participation in the SI.

During the interviews, primarily alumni (n = 39) spoke of the importance of networking at the SI. That said, many respondents were well aware of the networking opportunities at the SI, and, through the interviews, expressed multiple opinions about the potential value of networking. As respondent #59 explained, the appeal of networking at the SI was a way to enhance career development.

“*I thought this might be a good way for me to make some connections… with peers and mentors or whomever it may be. I just had a lot of hope that I would find some way to make a career for myself that was somehow still connected to global health*.”

Three respondents did not feel they gained much from the networking opportunity: two expressed regret at not making more of the opportunity (#18, #106), while the third felt that the opportunity was good but only of “*marginal benefit*” (#238). However, for the majority of respondents who spoke about networking, their expectations of participation in the SI were met. Respondent #137 expressed it thus:

“*There was a willingness to share, to form networks and links. So that I would say at the level of networking, it was a great experience*”.

#### Decreasing isolation through networking

While some interconnections were evident in the pre-SI phase, global health research is a highly dispersed field with researchers housed in many different departments and a variety of institutions where the primary focus may or may not be global health. Respondents, #137, #203, #113, and #73 all spoke of networking at the SI as a way to break out of professional isolation. The SI provided a space where researchers with similar interests could meet and share.

“*For me it’s important to get to know other people from around the world who are also possibly working on the same topic or are interested in the same topic, but are isolated. The idea then is to know what other people are doing and then, that maybe there is the possibility to join efforts and work together.*” (Respondent #137).

#### Meeting new people, strengthening existing connections

The SI presented a networking opportunity where participants could both meet new people and strengthen existing connections. Respondent #71 expressed these ideas, saying:

“*The most important thing is the partnership that developed as a result of the SI*” and that “*The connections and some of the networking with the French-language researchers was very useful and an experience I never would have otherwise had.*”

Respondent #103 gained access to a new group of individuals through the SI:

“*In my work in* [region]*, my relationships tended to be at the community level, but not at the researcher level. So that added a whole new layer of relationship and understanding and respect.*”

For some, networking represented a way to learn about the players and initiatives in the field of global health. Respondent #129 expressed this ‘generalist approach’ to networking. This respondent wanted to

“*…meet people and learn about what is going on in terms of other projects happening, especially between Canada and other countries.*”

Similarly, respondent #15 explained that:

“*…the connections formed were helpful for me to understand what kinds of global health things were happening in the world and how Canada was connected to them.”*

Other respondents articulated a more targeted motivation for networking, and connecting with other participants based on perceived similarities. For example, similar backgrounds or research interests. Respondent #82 reported connecting with people who spoke the same language and had similar research backgrounds. One FIT wanted to:

“*…enter into contact with an extensive network of researchers in circumstances similar to my own.*” (Respondent #94).

Respondent #113 shared a similar perspective:

“*In terms of networking with people that are close to my stage, it was also very beneficial in terms of information sharing and developing personal networks of young global health researchers.*”

In some cases, there was an interest in connecting with people based on perceived ‘differences’. For example, a FIT and an alumnus both spoke about the importance of establishing relationships with the “*younger generation*”. Respondent #94 framed this idea by commenting that:

“…*it is important to establish working relationships with young people who are beginning and have an interest in the types of work we do*.”

Similarly, many junior researchers noted the opportunity to connect with more experienced researchers from varied backgrounds. For instance, Respondent #102 expressed that:

“*…as an early career* [health researcher]*, I very much look forward to expanding my knowledge while working with diverse individuals from wide-ranging backgrounds, who are able to contribute different insights.*”

The social dimension of networking was expressed over and over as a central component of the SI. Respondent #73 expressed this idea by saying:

“*I think one of the big things about global health is the need to develop relationships and partnerships and trust among your collaborators. So having the time to dine together and socialize and talk about family and friends I think is a big part of developing a partnership. And so, I keep in touch with many of my colleagues and whenever there is a potential for linking up our work, with a good base at least you can explore that. And I do that with many of my friends from both the SIs.*”

Similarly, respondent #136 stated:

“*The biggest thing was the chance to talk and engage with other participants and facilitators.*”

“*People relax and actually talk to each other when you have a dinner together and I think that is important… it allows people to form bonds.*” (Respondent #121).

Channels were opened for the flow of human and social capital, defined as the knowledge and skills possessed by an individual, as well as the productive activity resulting from relationships between individuals [18]. Respondent #24 explained:

“*I was able to meet individuals from* [country] *that I kept in touch with, people that I had not known before, who I was able to share project ideas with, so that’s one immediate benefit.*”

Respondent #15 met several researchers who influenced her subsequent social connection and professional activities:

“*We actually became good friends because of those times. I learnt a lot more about her research which is in priority setting, I’ve read her stuff because of it, and it’s come out while I’m doing some teaching now.*”

Respondent #113 expressed similar sentiments:

“*It was important for networking… The academic relationships that developed were most useful because my department wasn’t focused on global health research. The SI helped bring focus to my research and link me in to other global health researchers in Canada at a professional level and at a graduate student level that I otherwise would not have tapped into.*”

#### Maintaining and using contacts

The movement and exchange of knowledge, opportunities, and resources continued in the post-F2F phase, through the maintenance and use of the connections established during the SI F2F session. Table 2 summarizes the quantitative phase I data on the extent to which respondents were still connected with other SI participants. The two categories are not mutually exclusive – participants were asked to both report who they are in contact with, as well as who they are actively collaborating with. The results presented in Table 2 present a snapshot in time of the connections and collaborations among SI participants. The results from the phase II interviews provided a richer understanding of the ebb and flow of connections between SI participants.

**Table 2:** Contact and collaboration among SI participants [Number (%)], phase I data

**The respondent in contact/collaboration with:**	**Nature of contact/collaboration:**	**Respondent (by role in SI)**		
		**Alumni (n = 36)**	**FITs (n = 8)**	**Facilitators-organizers (n = 17)**
Dyad-triad partner	In contact	27 (75%)	6 (75%)	Not asked
	Currently collaborating*	**16 (44%)**	**5 (63%)**	**Not asked**
Other participants in same summer institute year	In contact	16 (44%)	5 (63%)	5 (29%)
	Currently collaborating*	**6 (17%)**	**2 (25%)**	**4 (24%)**
Facilitators in same summer institute year	In contact	16 (44%)	6 (75%)	12 (71%)
	Currently collaborating*	**14 (39%)**	**6 (75%)**	**7 (41%)**
Other summer institute alumni	In contact	9 (25%)	4 (50%)	6 (35%)
	Currently collaborating*	**8 (22%)**	**1 (13%)**	**2 (12%)**
Other CCGHR members	In contact	21 (58%)	7 (88%)	13 (77%)
	Currently collaborating*	**12 (33%)**	**3 (38%)**	**3 (18%)**
None of the above	In contact	1 (3%)	0	1 (6%)
	Currently collaborating*	**8 (22%)**	**0**	**11 (65%)**

Non-bold values: past participants who are in contact; Bold values: past participants who are currently collaborating; *e.g., research projects, advisory boards, consultants, mentoring, organizing workshops, and capacity development activities.

The majority of respondents who mentioned still being in contact with other SI participants tended to have close contact with a small number of people. This paints the picture of tighter clusters within the broader SI network. Interestingly, respondents view this feature in both positive and negative lights. Respondent #103 took a positive view:

“*The other point from the SI experience is how wonderful it is to get to know colleagues from all over the world who are working on these issues out there and you know I’ve remained in contact with a couple of them and we’ve had interactions and that’s been excellent.*”

While respondent #106 presented a more negative perspective:

“*I keep in touch with certain persons but not as much as I expected. They were a lot of people in the institute, and finally I just keep in touch with two or three persons, and that is not enough.*”

As part of the SI program, facilitators were encouraged to maintain contact with their assigned dyad/triad after the F2F session, to continue supporting SI project development or implementation. Several facilitators spoke about being in contact with their dyad/triad, other alumni and/or facilitators in the weeks or months immediately after the F2F session, but in many cases these interactions tapered off over time.

The potential for collaboration surfaced repeatedly as an opportunity arising from networking at the SI. As Respondent #109 explained:

“*I really wanted to network to know people that were main actors in this field and have opportunities to collaborate with them outside of the SI experience*.”

While respondent #91 shared:

“*I see a lot of potential for future collaborations. There are some people that I met at the SI that I periodically exchange emails with.*”

#### Networking outcomes

Respondent #73 spoke of a collaborative research project that developed out of meeting a new person during the SI program, as well as instructing opportunities at a university that were also a result of relationship developed at the SI. Similarly, respondent #82 was invited to teach in another SI participant’s department after meeting at their SI. This respondent also worked on a research project and became more heavily involved in CCGHR activities as a result of connecting with a facilitator at the SI. Respondent #83 met an individual at the SI, and together they collaborated on an arts-based output. While respondent #251 has “*remained in contact with a couple of people… and we have ongoing projects.*”

For respondent #71, the opportunity to strengthen the dyad partnership “*has led to research productivity and fruitful collaboration, which has benefited both members of the dyad.*” However, respondent #59 expressed a concern about the possible disparities in who benefits the most from this opportunity. This respondent, a Canadian-based researcher, felt that her/his dyad partner’s career probably did not benefit from the experience to the same extent.

### Strengthening the Summer Institute network

Overall, there were a range of suggestions for strengthening the SI F2F session, including suggestions relating to content and structure. Interestingly, there were no suggestions for altering the networking opportunities during the SI, only an interest in seeing these connections maintained after the F2F session (refer to subsection “Participants’ suggestions for strengthening the SI alumni network”). Many suggestions revolved around the use of communication technologies or social media to keep participants connected. Others related to methods for fostering a sense of community among SI participants, as a way of strengthening the network.

One of the challenges expressed was the time needed to maintain contacts. As respondent #106 explains:

“*…as I am in an academic environment, I don’t have enough time to invest to maintain contact. But I feel glad to know these people and to have their name and contact on the list of the participants. This is a good advantage”*.

Furthermore, respondent #15 felt that:

“*…being outside of Canada is a barrier to remaining connected, particularly with the core CCGHR leadership team, because there aren’t as many opportunities for face-to-face interaction.*”

#### Participants’ suggestions for strengthening the SI alumni network

•Organize Skype meetings (#103)

•Increase use of social media and networking sites such as LinkedIn (#24)

•Create an alumni listserv (#91, 136)

•Use of email rather than webspace (#136)

•Think of ways to better involve previous SI participants (#13)

•Send abstracts of SI alumni’s research to SI participants of later years (#16)

•Continue CCGHR efforts to support previous SI participants in attending [their] forums/conferences (#24)

•Organize more satellite meetings at conferences that past SI participants will likely attend (#206)

•Continue occasional/regular communication from CCGHR personnel or SI facilitator (#5)

•Make funding available to students so they could pursue research with their partners (#121)

•Support alumni to take a lead on creating a strategy to keep people more connected (#73)

•Promote a sense of belonging and identity as a CCGHR member as an incentive for on-going participation (#59)

## Discussion

**Figure 1:**
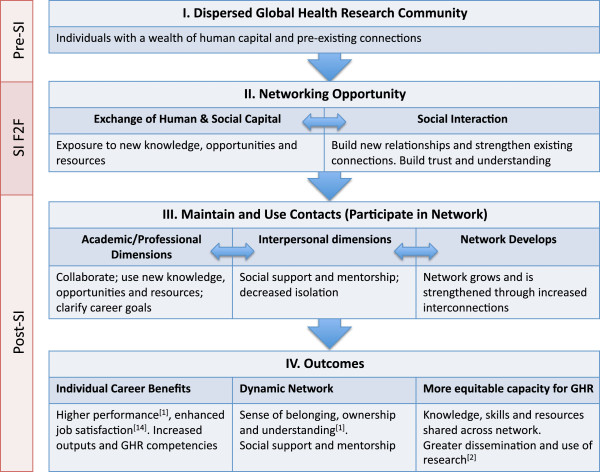
**Process model of networking within and across the SI phases.** SI: Summer Institute. F2F: Face-to-face session. GHR: Global health research.

We synthesized the literature and our findings from phases I and II of this research through the development of a new process model of networking across the SI phases (Figure 1).

### Making connections in the face-to-face (F2F) session

As in reports of other training programs with intensive sessions [19], deliberately creating a space for networking within the SI schedule allowed participants from a dispersed global health research community (box I, Figure 1) to strengthen existing connections and create new connections. Through these connections, new ideas, resources and opportunities were exchanged (box II, Figure 1). Informal social interaction played a critical role in forming professional connections and promoting exchange, as seen in Cross et al.’s [24] analysis of informal networks within organizations; their study concluded that “how work gets accomplished is increasingly reliant on the health of informal networks”. In our study, respondents emphasized the importance of socializing as a way to form strong bonds and long-term partnerships built on trust and fellowship formed through social interaction.

#### Persistence of connections

Networking existed within a larger context that included both the pre-SI F2F session and post-SI F2F session phases (represented by the left-hand column in Figure 1). In the post-SI phase, contacts needed to be used and maintained, or connections were lost (box III, Figure 1). Respondents most frequently reported sustained connections with only a select number of other participants, as did alumni of a South African masters’ program: “83% of full time respondents were in communication with their fellow students, but only 33% of part timers. The reason for maintaining contact was split between social (45%) and work/research related issues (36%)” [18]. Among SI participants, there would appear to be a loose network that can be tapped into over the long term, as evidenced by the way that participants spoke about partnerships, many of which did not develop until long after the F2F session.

It was largely up to the individuals to initiate and maintain the connections, both during the SI F2F session and post-SI period. Personality differences may affect the ease with which a participant in an intensive training engages in networking activities. Forret and Dougherty [13] found that gender, socioeconomic background, extraversion, self-esteem, and attitudes toward workplace politics all related to the networking behavior of managers and professionals. Some participants appeared to need a concrete, immediate reason to stay in contact while others seemed to be able to reconnect with fellow SI participants more easily.

### Outcomes of networking

As contacts were maintained and used during the post-SI phase, there were numerous potential outcomes impacting the individual and the broader field. The impacts on the individual were often concrete: benefiting from mentorship, clarification of career goals, access to new opportunities (research projects, fellowships, teaching opportunities, etc.), or the impacts could be less tangible, for example learning a new methodology, gaining respect for other researchers, or greater sense of belonging, ownership, and understanding [2] (left-hand cell of box IV, Figure 1).

There was some indication of perceived discrepancies in the extent to which SI participants benefited from the networking opportunities. One Canadian dyad partner felt her career benefitted more than her southern dyad partner’s career from networking at the SI. However, the data were too sparse to allow for deeper exploration of potential inequalities in using and benefitting from networking opportunities. Further research is needed to understand the facilitators and barriers, whether personal or contextual, that impact upon one’s ability to benefit from networking opportunities in global health research. Such facilitators and barriers could be embedded within the arrows of our process model (Figure 1) in its application to other intensive training opportunities in global health research.

### Maintaining a network of new global health researchers

Networking through the SI may also have impacts that extend beyond the individuals involved. The individual connections that formed through networking can be seen as the foundation of a dynamic network of global health researchers (box IV, Figure 1). It is through F2F or virtual interaction over time that networks develop trust and notions of reciprocity among their members [25]. These, in turn, can promote continued interaction and concrete collaboration among the SI participant members of the network. This network can then cross-link to existing networks in a dynamic process that Drimie and Quinlan [6] describe for research and policy networks. The simple act of networking among individuals can, over time, act as a force that shapes the structure and functions of the field of global health research (box IV, Figure 1), ideally towards more equitable and shared capacity.

When the respondents’ narrations of their experiences with networking are pieced together, a picture forms of the growth and development of a SI network. While it is evident from the phase I data and the phase II interviews that respondents remained in contact with each other and benefitted from professional relationships for their own career development, there was a desire among SI participants to strengthen this network, something Sub-Saharan African colleagues have noted among CARTA (http://www.cartafrica.org) doctoral fellows participating in Joint Advanced Seminars (Caroline Kabiru, personal communication).

The request for more structural supports to facilitate easier virtual networking and collaboration in the post-F2F phase is something with which CCGHR has grappled, via support for SI alumni meetings at conferences. At the Global Health Conference in Montreal, 2011, a call was issued for the creation of online profiles of global health researchers. An up-to-date database of global health researchers could enhance interaction and cross-pollination of ideas, and reduce duplication of efforts. A virtual networking platform as that which the CCGHR is currently developing could certainly enhance the ability to undertake collaborative, innovative, and creative global health research, as in a recent mentorship stories project involving several SI alumni and facilitators (http://www.ccghr.ca/working-groups/mentorship/).

### Limitations

Our response rates at each phase were not ideal, despite extensive efforts made to recruit participants from both Canada and LMICs. The language of response indicated that the majority of interviews were conducted in English, not fully reflecting the mix of languages among SI participants. Further, we did not have the appropriate data for a social network analysis through which we might have drilled down upon structural features of relationships in the SI network, the type of information exchanged between individuals, the perceived level of impact of the exchanged information knowledge transfer [26], and/or decision-making [24]. As the current research was exploratory, further directed research on networking among new global health researchers, its genesis, and its maintenance would be worthwhile, building upon our process model.

## Conclusions

As evidenced through this exploration of participants’ experiences of networking at the CCGHR SIs, networking as part of global health capacity strengthening initiatives can have an important impact on new global health researchers. It can foster the building of long-term professional relationships and open channels for the exchange of knowledge, resources, and mutual support, particularly among researchers with common, specific interests from different parts of the world. Consciously designing networking opportunities as part of F2F and virtual intensive, training sessions has great potential for leveraging positive outcomes among new global health researchers.

Further research is needed on mechanisms involved in the successful formation and maintenance of contacts and the facilitators of and barriers to benefiting from networking opportunities. A deeper understanding of the diversity of experiences across countries that is sensitive to the varying experiences of global health researchers in different contexts is essential to both building strong, inclusive networks of global health researchers and elaborating upon our networking process model.

Institutional supports are needed to create robust, dynamic networks accessible to health researchers around the world and sustainable over the long term [7]. Developing such supports pose a challenge to NGOs’ like the CCGHR and COHRED, which aim to sustainably and equitably develop health research capacity for development, as well as to funders of health research capacity strengthening, in both high income countries and LMICs. Intentional inclusion of networking resources and opportunities, building on virtual platforms and social media, could become as mainstream for capacity strengthening as fellowships and travel awards are now. We look forward to other colleagues sharing experiences of structuring intentional networking opportunities and evaluating their impacts among new global health researchers.

## Abbreviations

CCGHR: Canadian coalition of global health research; F2F: Face-to-face; FITs: Facilitators-in-training; LMICs: Low- and middle-income countries; NGO: Non-governmental organizations; SI: Summer institute for new global health researchers; SI-LEAD: SI alumni leadership program.

## Competing interests

The authors declare no competing interests.

## Authors’ contributions

LL was involved in research project design, data collection, data analysis and is the lead author of this manuscript. DCC was involved in the research project conception, data collection and analysis and in making major contributions to this manuscript. PGR was involved in coordinating and data collection, data analysis, report writing as well as making major contributions to this manuscript.

## Supplementary Material

Additional file 1 Networking analysis framework for phase II interview transcripts. Click here for file

Additional file 2 Major categories in the phase II interview. Click here for file
