# Supplementary material for: Networking among young global health researchers through an intensive training approach: a mixed methods exploratory study
Source: Health Res Policy Syst. 2014 Jan 25;12:5. doi: 10.1186/1478-4505-12-5 (PMC3916077; doi:10.1186/1478-4505-12-5)
Supplement: Additional file 1 — Networking analysis framework for phase II interview transcripts. [file 1478-4505-12-5-S1.doc]

| **Additional File 1** - Networking Analysis Framework for Phase II interview transcripts | | |
| --- | --- | --- |
| Query | Analysis | Data presentation |
| Networking node | Thematic analysis following the basic structure:   - Types of networking described - The processes and features that both facilitated or hindered networking at the SI - Immediate and long-term implications and impacts of engaging (or not) in networking - Overarching ideas or commentary on the connection of networking to global health research, collaboration and capacity development. | Narrative of thematic analysis |
| Suggestions for change nodes | - Categorization of different types of “suggestions for change” - Compilation of specific details/suggestions relating to networking and networks. - Apply insights from this section to conceptual framework: facilitators and barriers to networking and participating in a network, as well as how networking and networks can be strengthened in the face-to-face and follow-up periods. | Narrative of suggestions, intertwined  Inform model/  framework development |
| Q1 b) How did you learn about and become involved with the SI? | - Pull question from NVivo and categorize responses - Apply insights to model/framework to inform nature of connections/network prior to participation in the SI. | Include examples to help explain conceptual model |
