# Supplementary material for: Networking among young global health researchers through an intensive training approach: a mixed methods exploratory study
Source: Health Res Policy Syst. 2014 Jan 25;12:5. doi: 10.1186/1478-4505-12-5 (PMC3916077; doi:10.1186/1478-4505-12-5)
Supplement: Additional file 2 — Major categories in the phase II interview. [file 1478-4505-12-5-S2.doc]

**Additional File 2**. Major Categories in the Phase II Interview

1. Respondent background: Education/professional background, disciplinary background, work experience, methodological skills (qualitative and quantitative, heath systems and policy, etc.) familiarity with various teaching styles, etc.;
2. SI Project Summary: The topic and types of research in which they were involved and on which they developed the SI Knowledge Translation Plan during the SI;
3. SI & CCGHR Components: the extent of involvement/experience with various components of the SI as well as the broader CCGHR activities (e.g., SI preparation, SI follow up, FITs Project, SI-LEAD, SI planning, etc.);
4. SI Expectations; The expectations the respondents had when applying for the SI in terms of knowledge gains, skills development, networking, consultation on career advice, etc.;
5. SI Feedback: Specific feedback on the structure and content of the SI, including the Pre-SI email communications, face-to-face sessions and post-SI follow up activities. Respondents were asked for constructive feedback on how the SI could be improved to better meet the needs and expectations of the participants;
6. Research Outputs: information about peer-review publications, policy briefs, conference presentations and other types of dissemination strategies that was associated with their SI participation;
7. Influencing Factors: Enabling and impeding factors that influence the ability to succeed in conducting health research and developing a career in global health research; and
8. Career Trajectories: Information on current work and responsibilities, ongoing or new collaborations with other SI alumni or SI facilitators and the potential role of the SI in their career development.
